# New products

**DOI:** 10.1155/S1463924601000128

**Published:** 2001


Journal of Automated Methods & Management in Chemistry, Vol. 23, No. 3 (May–June 2001) pp. 93–96

### New products

### Econofil—syringe filters for HPLC sample preparation

When preparing samples for injection, syringe filters give convenient, rapid assurance that your column and injector will not be damaged by particulates.

Econofil syringe filters are the cost-effective means of preventing column blockage and injector wear.

Filter discs of 4, 8, 13 and 25 mm diameter are available to deal with the range of samples used in analyses from extremely small volumes of sample from a micro-syringe up to many millilitres of formulations.

Standard 0.45 µm porosity filters are complemented by 0.2 µm porosity filters. These are available to give removal of extremely fine particulate materials.

Econofil have a comprehensive range of filters for aqueous, organic and mixed sample clean-up.

Organic clean-up may be carried out with Econofil PTFE and PVDF membranes.

For general HPLC solvent and aqueous mobile phase clean-up, Econofil Nylon and PVDF offer the widest applicability.

Econofil syringe filters are available in boxes of 100 or 500 units. The sealed bodies are of polypropylene, selected for cleanliness and low extractability.

The unit cost with these filters are lower than with almost any equivalent syringe filters.

*For further information call + 44 (0)1707 394949 or visit http://www.chromacol.com*

### SAV-Pak—multi-purpose HPLC column protection

HPLC columns need protection from impurities both visible and invisible. The new SAV-Pak guard column system gives multiple protection of expensive analytical columns by being both a guard column and an in-line filter.

The SAV-Pak contains interchangeable parts that allow direct connection to the majority of HPLC columns without any need to modify the fittings. Fitting and charging of a range of Generic guard cartridges takes a matter of seconds to produce a leak-proof protection at the column inlet.

If concerned about particulates being introduced into the system, the same unit may be changed to hold a single disk filter to give effective in-line protection.

The guard cartridges and fittings are manufactured from PEEK to give strength and inertness.

SAV-Pak guards and filters are supplied in an easy-to-use kit with enough parts to construct one guard unit and one filter unit.

Guard cartridges are packed with C18, C8, CN, Amino and Silica.

*For further details please call Chromacol on + 44 (0)1707 394949 or visit http://www.chromacol.com. For more PR information please contact Charlie Cook at Chromacol on + 44 (0)1707 394949*

### Cost-effective evaporation: Genevac launches new centrifugal evaporator for general pharmaceutical laboratory applications

Genevac, the world leader in centrifugal evaporation technology, has recently expanded its popular high specification range of HT Series Bench-Top units with the launch of the DD-4.

Designed for general use in the pharmaceutical laboratory, the DD-4 is a cost-effective centrifugal evaporation system for solvent removal in drug discovery applications.

Not fitted with some of the high performance features in the HT Series Systems, the DD-4 is designed for fast, effective operation in less demanding laboratory applications. It features interchangeable, high performance aluminium and swings rotors (to accommodate samples in test tubes and microtitre plates) and an infra-red sample temperature control to ensure rapid evaporation at controlled temperatures.

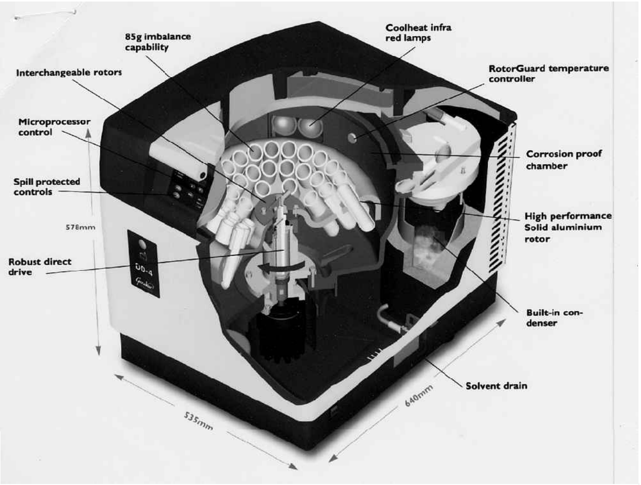

*Genevac DD-4 bench-top centrifugal evaporator.*

A number of optional Genevac features are also available, including the AquaSpeed system for rapid evaporation of aqueous samples, the Dri-Pure anti-bumping system and the unique CVP 100 Vapour Vacuum Pump for high performance evaporation, corrosion resistance and reduced maintenance.

*For more information about the Genevac DD-4 Evaporation System, please contact: Teresa Picas, Marketing Co-ordinator, Genevac Ltd, Farthing Road, Sproughton, Ipswich IP1 5AP, UK. Tel: + 44 (0)1473 240000; Fax: + 44 (0)1473 461176; e-mail: teresa.picas@genevac.co.uk; URL—http://www.genevac.co.uk*

**Metrohm's 792 Basic IC with chemical suppression—'ideal for teaching and basic ion chromatography applications'**

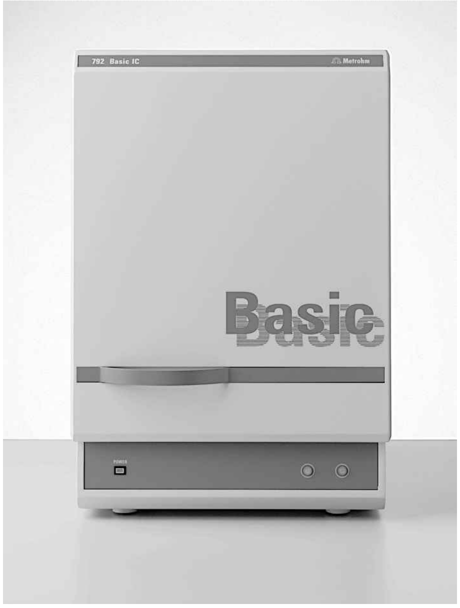

Where and how can ion chromatography be taught and learned? Up to now only complex or expensive solutions were possible. Metrohm ideally covers the ion chromatography teaching and training sectors with the new 792 Basic IC.

With the 792 Basic IC we would like to

- enable schools, universities, further and advanced training institutes as well as in-house training laboratories to acquire an extremely favourably-priced ion chromatograph;
- while at the same time we are publishing a textbook providing an illustrative introduction to the basic principles of ion chromatography in which the theory is explained both simply and at a more demanding level; it also contains 22 experiments, which are described in detail.

No sacrifice has been made with respect to the quality and flexibility of the system. The 792 Basic IC is a completely practical ion chromatograph constructed from time-proven Metrohm components:

- low-pulsation double piston pump—no external nitrogen or helium supply is necessary;
- purge valve;
- electric injection valve; electronic suppression for anions and cations;
- chemical suppression for anions with the 'MSM' Metrohm Suppressor Module;
- built-in 2-channel peristaltic pump;
- high-performance conductivity detector, temperature-stabilized at 40°C (better than ±0.01°C);
- thermally and electronically shielded housing;
- anion and cation operation;
- integral data acquistion and A/D-conversion;
- PC-control.

The 792 Basic IC is naturally not just limited to educational requirements. For standard applications in analytical laboratories it supplies reproducible and exact results within a very short time. The detection sensitivity is better than 100 ppb (0.1 mg/l).

For all Metrohm instruments, including the new 792 Basic IC, a further term has become increasingly important: the 'cost of ownership'. This covers the running costs associated with the instrument; with the Basic IC these are unusually low.

*For further information please contact Metrohm UK Ltd—Tel: +44 (0)12808 24824; Fax: +44 (0)12808 24800; e-mail: enquiry@metrohm.co.uk; URL—http://www.metrohm.co.uk*

**In-line ultrafiltration with the new 788 IC filtration sample processor**

In modern ion chromatography, it is recommended that the samples always be filtered before injection. Membrane filters with pore sizes of 0.45 µm or less are normally used for the filtration. However, the relatively high price of such disposable filters limits their use.

The new 788 IC filtration sample processor integrates membrane filtration as a sample preparation step directly into your IC system. Equipped with an ultrafiltration cell, it combines in-line filtration with automatic sample application.

The samples are placed directly on the turntable and are then processed completely automatically by the IC filtration sample processor. The sample solution to be filtered passes through a membrane. At the same time, the filtrate is aspirated off from the rear of this membrane, transferred to the sample injection valve of the ion chromatograph and then injected.

Depending on the sample matrix and its pore size and properties, the filtration membrane can be used for many or for only a few samples.

Ultrafiltration using the 788 IC filtration sample processor is particularly suitable for light-to-medium-loaded samples such as drinking water, surface water and waste-

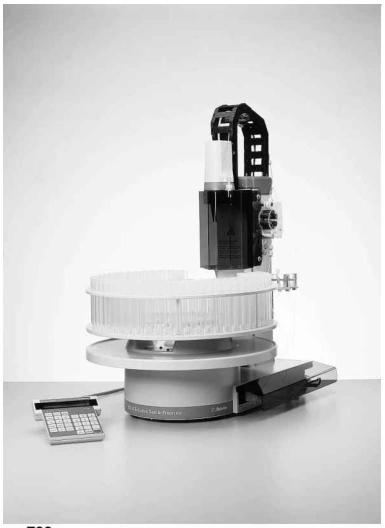

*The Metrohm 788 IC filtration sample processor.*

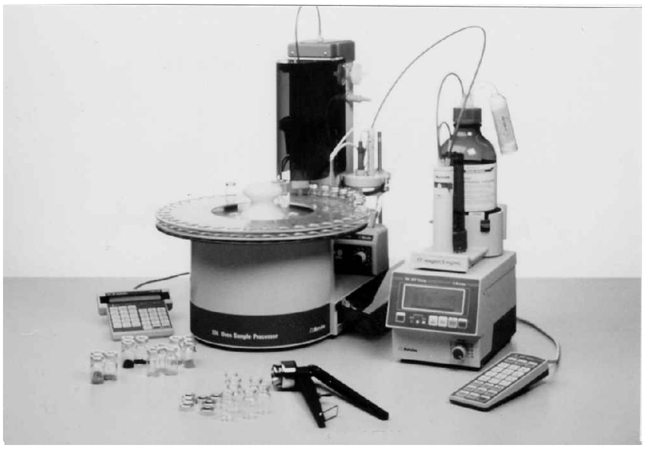

*Automated Karl Fischer moisture analysis system.*

water, digestion solutions, and extracts. Unloaded samples can be injected directly with the same sample changer, i.e. without filtration. With strongly-loaded samples, e.g. fruit juice containing fruit pulp, in-line sample dialysis with the 754 dialysis unit is recommended.

The 788 IC filtration sample processor can be used as the master or as a slave in a PC-controlled IC system. The sample rack offers places for 127 × 11 ml sample vessels. Two additional rinsing positions ensure carry-over-free sample application even with very different sample matrices.

*For further information please contact Metrohm UK Ltd—Tel: +44 (0)12808 24800; e-mail: enquiry@metrohm.co.uk; URL—http://www.metrohm.co.uk*

### Moisture content of heat sensitive pharmaceuticals

The 774 automated Karl Fischer system (the Metrohm 774 KF system) allows up to 32 samples to be determined for moisture content even if the sample is a heat sensitive pharmaceutical product. This is accomplished by using a special temperature gradient profile on each individual sample, thus allowing the sample to be heated gently. This profile technique also allows the determination of bound and unbound moisture within these products and produces a record of the 'determined kinetics of water release as a function of temperature'. Products whose moisture content has been historically difficult to determine, e.g. simple and complex amines, are easily and cost effectively determined using the Metrohm 774 KF system.

Freeze-dried products that are produced and presealed into vials of all sizes at the point of manufacture can be transferred to the 774 sample rack and from here the water content is determined without opening or transferring products into other vessels. This removes all problems of health and safety, e.g. allowing a product being open to the atmosphere, and of course removes all possible moisture contamination of the sample during transfer (which could cause errors in the moisture determination later on). Detection limits from 1 mg per litre up to 100% can be determined, with an analysis time of 3–5 minutes over a temperature range of 50–250°C.

Complete archiving of all data, including curves, calibration, calculations, methods, etc., are part of the 774 system, making it ideal for GLP, FDA, ISO and NAMAS auditing.

*For further information on this unique system please contact Metrohm UK Ltd—Tel: + 44 (0)12808 24824; Fax: + 44 (0)12808 24800; e-mail: enquiry@metrohm.co.uk; URL—http://www.metrohm.co.uk*

### Genevac develops new generation of Mega Evaporation Systems

Genevac has launched a new generation of its Mega Centrifugal Evaporation System.

The Series II Mega System provides ultra high throughput solvent evaporation with the same new features as are incorporated into Genevac's Series II HT Systems, such as built-in PC, 100 run memory, continuous running condensers, and pressure control with multi-stage runs.

### *High throughput and efficiency*

Mega Systems are designed to ease production bottlenecks with the added advantages of requiring less laboratory space, and less investment and maintenance than the use of conventional smaller systems. They incorporate a very large evaporation chamber up to 1.2 m diameter according to model—which is big enough to accommodate the large numbers of samples often required in a production environment.

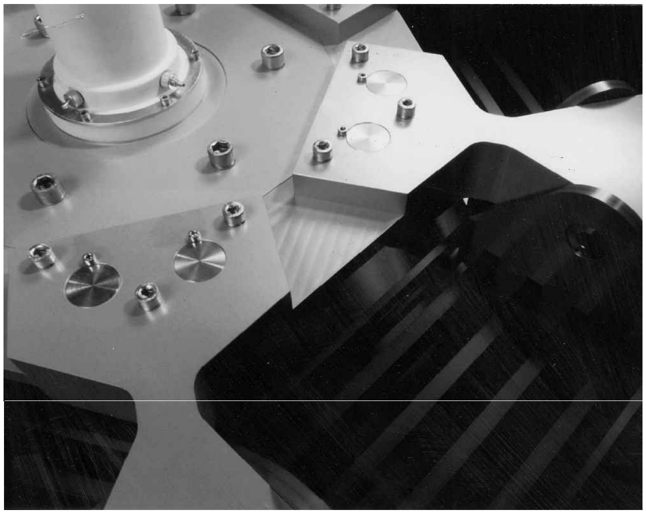

*Mega series II drug discovery system.*

*Format flexibility*

Accommodating samples in an extensive variety of microtitre or smaller formats, Mega Systems can also handle larger formats, such as fraction collector racks from LC/MS systems, larger reaction blocks or proprietary units

*For more information about the Genevac Series II Mega System, please contact: Teresa Picas, Marketing Co-ordinator, Genevac Ltd, Farthing Road, Sproughton, Ipswich IP1 5AP, UK. Tel: + 44 (0)1473 240000; Fax: + 44 (0)1473 461176; e-mail: teresa.picas@genevac.co.uk; URL—http://www.genevac.co.uk*

### Zymark and QIAGEN create an ultrahigh throughput nucleic acid handling platform

Zymark Corporation and QIAGEN N.V. have announced that they have entered into a strategic alliance addressing the use of ultrahigh throughput sample and liquid handling automation. The organizations will develop and launch systems that combine QIAGEN's BioRobot™ platforms with Zymark's RapidPlate™ 96/384 Microplate Pipetting Workstation, Zymark's Staccato™ Applications Focused Workstations and Zymark's Twister™ Universal Microplate Handler. The resulting systems will satisfy the increasing need in the genomics marketplace for flexible, easy-to-use ultrahigh throughput nucleic acid handling platforms. QIAGEN chose to standardize with the Zymark liquid handling and robotic technologies.

QIAGEN N.V., a Dutch holding company, is one of the world's leading providers of technologies and products for the separation, purification and handling of nucleic acids. Zymark is one of the only manufacturers in the world concentrated exclusively on laboratory automation and robotic solutions for the rapidly expanding life sciences marketplace. It also hosts the annual ISLAR Conference on Laboratory Automation.

*For more information contact Lynda J. Thomas, Marketing Communications, Zymark Corporation of Hopkinton, MA, USA. Tel: + 1 (0)508 497 6549; e-mail: lynda.thomas@zymark.com; URL—http://www.zymark.com; or QIAGEN: URL—http://www.QIAGEN.com*

